# Research on the innate immune response in transgenic mice following ischemic stroke

**DOI:** 10.3389/fnagi.2024.1476913

**Published:** 2024-11-22

**Authors:** Chao Yuan, Yuting Shentu, Qiuhong Ji

**Affiliations:** ^1^Department of Neurology, Nantong University, Nantong, China; ^2^Department of Neurology, Affiliated Hospital of Nantong University, Nantong, China

**Keywords:** ischemic stroke, transgenic mice, innate immunity, microglia, macrophages

## Abstract

The high incidence, death, disability, and recurrence of ischemic stroke (CIS) place a significant cost on families and society. According to recent research on the condition, immune-related damage is a major contributor to the development and occurrence of CIS. Innate immunity and adaptive immunity are the two primary categories of the immune system in the body. The body’s first line of defense is innate immunity, and immune cells play a role in every stage of the immune system. At the same time, protein molecules play a vital function in regulating and differentiating immune cells. It can be said that protein molecules are the foundation of immune regulation. Model mice are necessary for us to examine fixed compounds in our studies. Conditional deletion and overexpression mouse models are the two primary categories of model mice. Numerous model mice have been documented in CIS research. The study of innate immune responses following ischemic stroke will benefit more from the use of these transgenic mice that target innate immunity. This paper analyzes the literature on transgenic mice related to innate immune responses following ischemic stroke because of the significance of these responses. It is anticipated to produce novel medications, improve clinical treatment guidance, and undergo a metamorphosis and application in the clinic in the future.

## 1 Research background

The term “ischemic stroke” describes a clinical state marked by the abrupt development of neurological impairments brought on by localized brain tissue hypoxia necrosis and ischemia brought on by a variety of cerebrovascular illnesses that impede the brain’s blood supply. It puts a significant strain on families and society due to its high rates of occurrence, death, disability, and recurrence (Wu et al., 2019; Li et al., 2022; Luo et al., 2024).

The term “innate immunity” describes the body’s basic physiological defense mechanisms, which include the ability to mount a suitable immune response in response to the invasion of numerous pathogens and other substances (Abbas et al., 2012). Innate immune cells include neutrophils, monocytes, macrophages, mast cells, and dendritic cells, as well as specific lymphocyte populations, such as natural killer (NK) cells and gamma-delta T (γδT) cells (Abbas et al., 2012).

The innate immune system plays a crucial role in both the acute phase and the recovery phase of ischemic stroke (Xiong et al., 2016; Li et al., 2021; Lee et al., 2004; Colton, 2009; Price et al., 2004; Garcia-Bonilla et al., 2014; Gidday et al., 2005; Gelderblom et al., 2009; Jin et al., 2009). Microglia/macrophages are the primary resident innate immune cells in the brain. Macrophages and microglia are quickly activated following an ischemic stroke, with microglia being more important in the initial phases (Xiong et al., 2016). Microglia/macrophages release the production of pro-inflammatory cytokines, including TNF (tumor necrosis factor) and IL-1 (interleukin-1), reactive oxygen species (ROS) and reactive nitrogen species (RNS), as well as proteases such as MMPs (matrix metalloproteinases). In the early stages, microglia can harm blood vessels, astrocytes, neurons, and oligodendrocytes (Li et al., 2021; Lee et al., 2004; Colton, 2009). Neutrophils play a crucial role in the inflammatory response, being the most abundant type of white blood cells to first infiltrate from the bloodstream into the brain parenchyma (Price et al., 2004; Garcia-Bonilla et al., 2014; Gidday et al., 2005; Gelderblom et al., 2009; Jin et al., 2009). By producing metalloproteinases and reactive oxygen species and by activating their own inducible nitric oxide synthase (iNOS), neutrophils contribute to the inflammatory response. This results in endothelial cell membrane damage and increased blood–brain barrier permeability, which makes it easier for monocytes and macrophages to infiltrate the injured area of the blood–brain barrier and expand the area of injury after infiltration (Price et al., 2004; Garcia-Bonilla et al., 2014; Gidday et al., 2005; Gelderblom et al., 2009; Jin et al., 2009). Microglia play an important role in the later stages of the repair process. In the later stages, due to the release of transforming growth factor β1 (TGF-β1), glial cell line-derived neurotrophic factor (GDNF), and anti-inflammatory interleukin-10 (IL-10) by microglia, they may exert protective or reparative effects (M2 phenotype) (Li et al., 2021; Lee et al., 2004; Colton, 2009).

**Figure 1:**
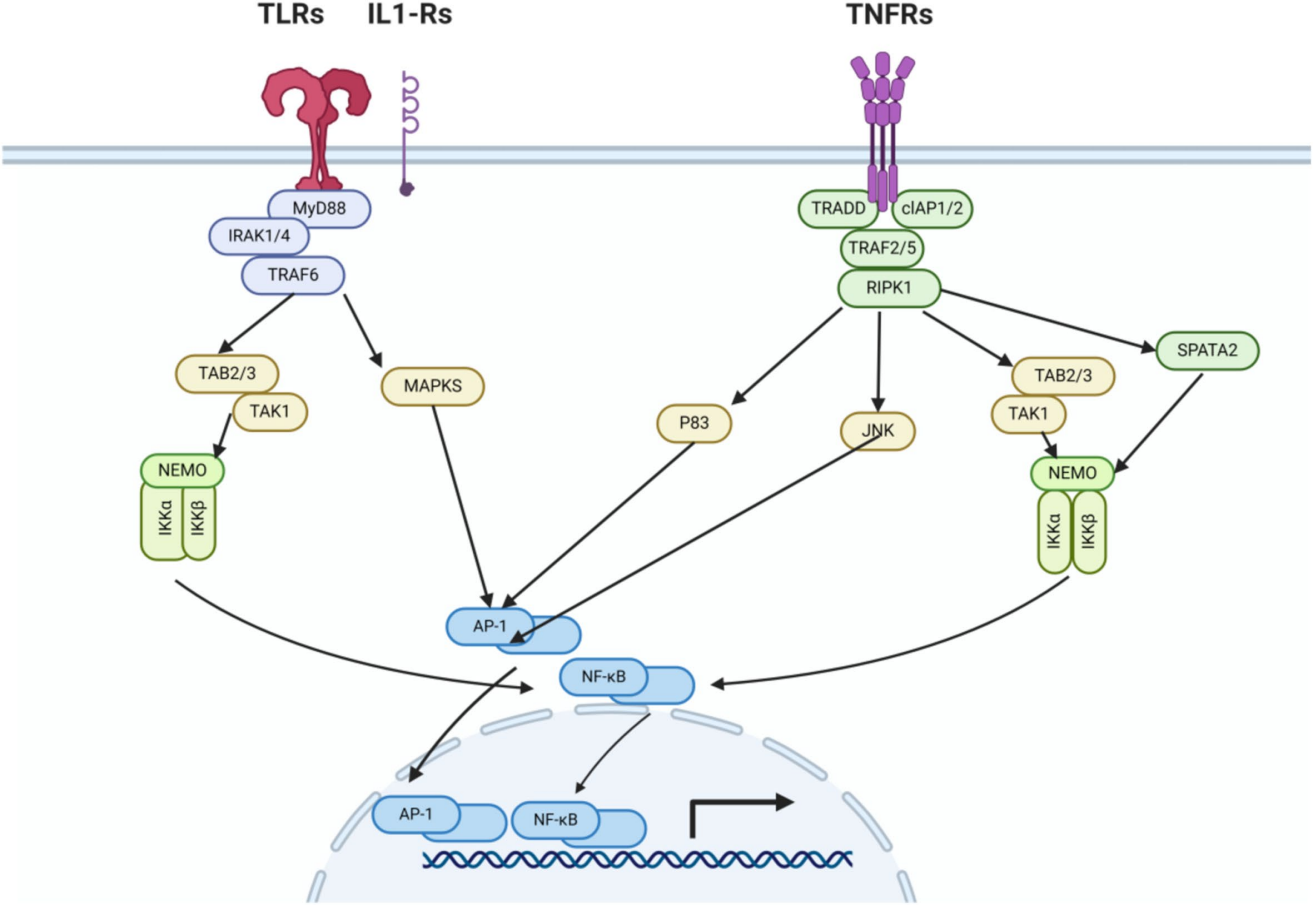
Innate immune signaling pathways of TLRs, IL1-Rs, and TNFRs. After cerebral ischemia–reperfusion injury, the release of DAMPs leads to the activation of TLR receptors. Intracellular pathways (MyD88, TRAF6, TAK1/MAPKs, NF-κB/AP-1) activate transcription factors, promoting the production of chemokines, cytokines, and reactive oxygen species, as well as the extravasation of leukocytes from the circulation, thereby exacerbating cellular damage. The production of cytokines triggers the activation of their corresponding receptors, for example, TNFα activates the TNF receptor and IL-1 activates the IL-1 receptor. These receptors then activate transcription factors via intracellular pathways, intensifying the damage to cells.

Within the brain, microglia and leukocytes have pattern recognition receptors on their cell membranes. After cerebral ischemia, damaged neurons release a large number of damage-associated molecular patterns (DAMPs). These receptors can be activated by DAMPs (Schilling et al., 2003; Richard et al., 2017; Sadat-Hatamnezhad et al., 2016; Downes et al., 2013), and the activation of these receptors triggers a series of processes leading to the expression of pro-inflammatory genes (Jain and Pasare, 2017; Masuda et al., 2011). The transcription factor NF-κB (nuclear factor kappa B) triggers the expression of genes encoding pro-inflammatory molecules, including IL-1β (interleukin-1 beta), IL-6 (interleukin-6), IL-8 (interleukin-8), and TNF-*α* (tumor necrosis factor alpha). Another transcription factor involved in the activation of pro-inflammatory gene expression is AP-1 (activator protein 1), which is an important activator for the expression of IL-1 and TNF-*α* (Xiong et al., 2016; Wang et al., 2018; Li et al., 2019; Xie et al., 2020) (Figure 1).

**Figure 2:**
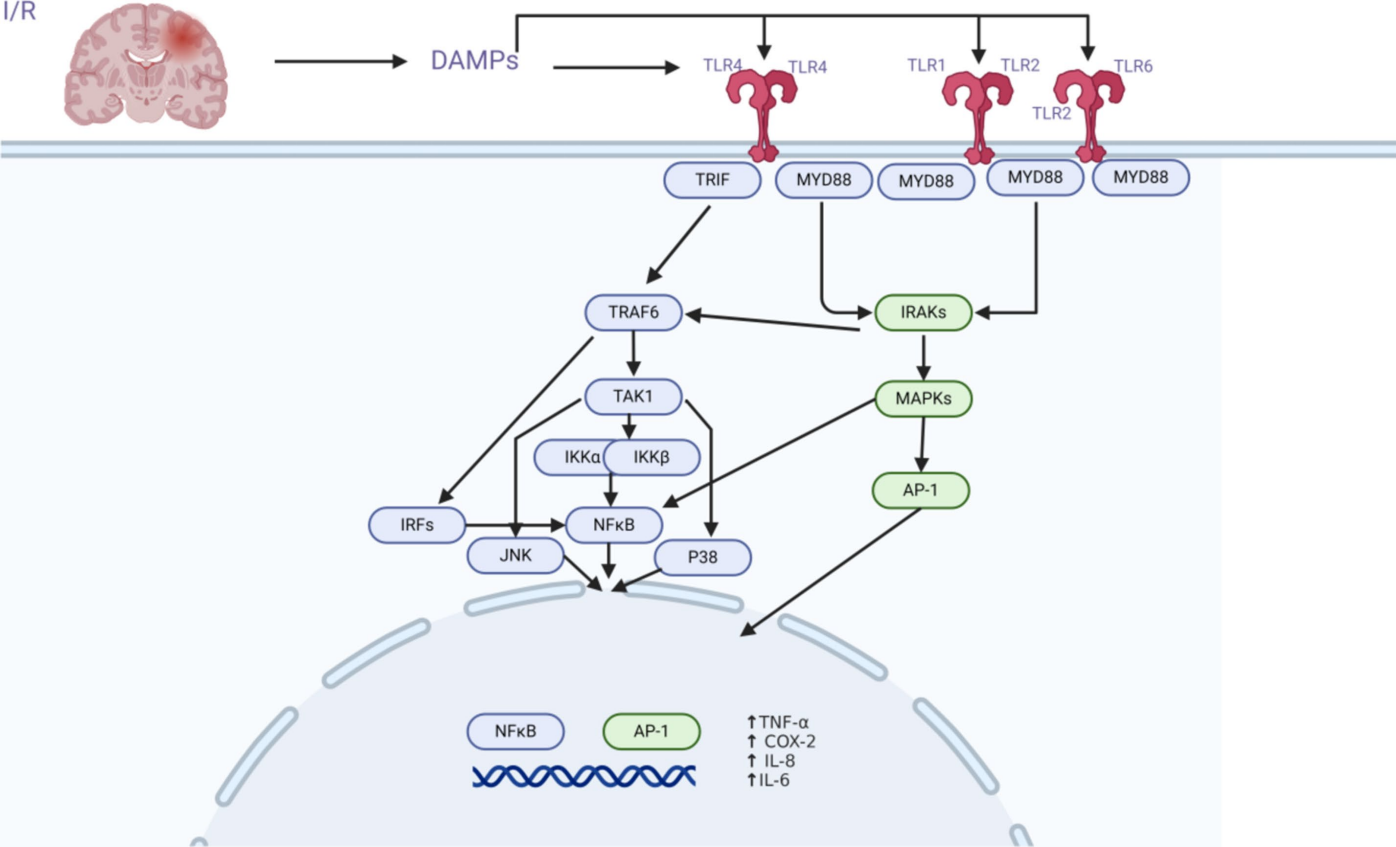
Schematic of the TLR2 and TLR4 signaling pathways. TLR2 and TLR4 are important players in the complicated process of cerebral ischemia–reperfusion damage. Following brain ischemia–reperfusion injury, TLR2 and TLR4 receptors become active due to the generation of DAMPs. Through intracellular pathways (MyD88/TRIF, TRAF6, TAK1/MAPKs, NFκB/AP-1), transcription factors are activated, resulting in the production of cytokines and chemokines, which play a crucial role in the modulation of the innate immune response to cerebral ischemia–reperfusion.

TLR2, TLR4/MyD88/NF-κB, and AP-1 are important signaling pathways in the innate immune response to ischemic stroke. The TLR2 and TLR4 receptors primarily signal through the MyD88 protein (myeloid differentiation primary response protein) to activate the transcription factors NF-κB and AP-1, triggering the expression of genes encoding pro-inflammatory molecules (Xiong et al., 2016; Zhu et al., 2018; Fitzgerald et al., 2001; Liu et al., 2023) (Figure 2).

Researchers working in the field of neurology have utilized transgenic mouse technology to get additional insight into the innate immune response mechanisms of the central nervous system after an ischemic stroke. The development of numerous genetically engineered mice that have had particular genes overexpressed or knocked out has made it feasible to investigate the molecular pathways underlying the innate immune response to ischemic stroke. This article discusses transgenic mice that target innate immunity cells, innate immune chemicals, pattern recognition receptors, and associated signaling pathways. These topics may have implications for therapeutic applications, the creation of novel drugs, and improved clinical treatment recommendations in the future. The research on the innate immune response in transgenic mice after ischemic stroke is thoroughly discussed in this article.

## 2 Research methods

### 2.1 Search strategy

Under the guidance of the PRISMA framework, we conducted a systematic literature search to identify publications related to transgenic mouse studies on innate immune responses following ischemic stroke.

Using PUBMED on October 18, 2024, the search query was formulated as follows:(“stroke”[MeSH Terms]) AND (“transgenic mice”[MeSH Terms]).

All records were limited to English language publications.

### 2.2 Eligibility criteria and search results

From PUBMED, a total of 1,501 records were retrieved. After removing duplicates, 1,368 records were screened. Of these, 1,299 records were excluded because they did not pertain to studies on transgenic mice and the innate immune response in ischemic stroke. In total, 69 studies met the eligibility criteria for review.

## 3 Innate immune response following ischemic stroke

### 3.1 Modulating the innate immune response following ischemic stroke by targeting microglia, neutrophils, mononuclear macrophages, and mast cells

#### 3.1.1 Microglial IRF5-CKO mice and microglial IRF4-CKO mice

Microglia are the resident immune cells of the brain and play a central role in initiating and maintaining immune responses. After pathogenic stimulation, they can adopt two activation states, leading to either a pro-inflammatory (M1) or an anti-inflammatory (M2) phenotype (Al Mamun et al., 2020). Interferon regulatory factors (IRFs) mediate the activation of peripheral immune cells and macrophages in other inflammatory diseases. Interferon regulatory factors (IRFs) mediate the activation of peripheral immune cells and macrophages in other inflammatory diseases. A regulatory axis that balances the pro- and anti-inflammatory activation of microglia is formed by the IRF5-IRF4 signaling pathway (Al Mamun et al., 2020). After ischemic stroke, microglia exhibit both pro-inflammatory and anti-inflammatory responses (M1–M2 phenotypes) that coexist and antagonize each other. In microglial IRF5 CKO mice, the absence of the IRF5 signal leads to increased expression of IRF4, enhanced M2 activation, reduced pro-inflammatory response, and improved outcomes in ischemic stroke (Al Mamun et al., 2020). Because IRF4 signaling is absent in microglial IRF4 CKO animals, there is increased IRF5 expression, higher M1 activation, impaired pro-inflammatory responses, and decreased functional recovery (Al Mamun et al., 2020).

#### 3.1.2 Microglial: innate immune response in TREM2-KO mice following ischemic stroke

After ischemia, phagocytosis of cellular debris is essential for reducing inflammation and reestablishing tissue homeostasis. Triggering receptor expressed on myeloid cells 2 (TREM2), expressed on bone marrow cells, is a microglial surface receptor that transmits intracellular signals through the adaptor protein DAP12. In an anti-inflammatory environment, it can clear apoptotic material through phagocytosis (Sieber et al., 2013).

After ischemic stroke, TREM2-KO mice exhibit reduced microglial activity, with decreased transcription of pro-inflammatory cytokines TNFα, IL-1α, and IL-1β, as well as reduced transcription of chemokines CCL2 (MCP1), CCL3 (MIP1α), and chemokine receptor CX3CR1. Consequently, the infiltration of CD3-positive T cells is diminished. TREM2-KO mice exhibit a reduced subacute inflammatory response to ischemic stroke, whereas the infarct size remains unchanged (Sieber et al., 2013).

#### 3.1.3 Microglia/macrophages: innate immune response in microglia/macrophage vitamin D receptor CKO mice following ischemic stroke

Vitamin D deficiency is associated with an increased risk of various diseases and adverse outcomes, including cardiovascular diseases, cancers, autoimmune diseases, and neurological disorders (Cui et al., 2023). There is a significant association between vitamin D deficiency and adverse cerebrovascular events, including an increased risk of ischemic stroke and poor stroke prognosis (Cui et al., 2023).

Following ischemic stroke, microglia/macrophage VDR-CKO mice exhibit an enhanced M1 phenotype in microglia/macrophages, with substantial secretion of TNF-*α* and IFN-*γ*. The blood–brain barrier is disrupted, peripheral T lymphocyte infiltration is enhanced, and CXCL10 is released from endothelial cells as a result of these inflammatory cytokines, aggravating ischemic brain injury (Cui et al., 2023).

#### 3.1.4 Microglia: innate immune response in NHE-1^+/−^ mice following ischemic stroke

The activation of microglia relies on the Na^+^/H^+^ exchange-mediated H^+^ homeostasis; preventing the accumulation of H^+^ and cytosolic acidification is crucial for maintaining the respiratory burst activity mediated by NADPH oxidase in microglia (Shi et al., 2011).

NHE-1 (sodium-hydrogen exchanger isoform 1) ^+/−^ mice show improved neurological and functional prognosis following ischemic stroke, as well as decreased pro-inflammatory responses, decreased NADPH oxidase activity, and lessened microglial activation (Shi et al., 2011).

#### 3.1.5 Microglia: innate immune response in microglial NKCC1 KO mice following ischemic stroke

Members of the plasma membrane cation-chloride cotransporter (CCC) family, such as the neuron-specific K^+^/Cl^−^ transporter KCC2 and the ubiquitously expressed Na^+^-K^+^-2Cl^−^ cotransporter NKCC1 (encoded by the Slc12a2 gene), are increasingly the focus of research in central nervous system (CNS) diseases, including neuropsychiatric disorders, epilepsy, stroke, and dementia (Tóth et al., 2022).

Microglial NKCC1 KO mice show enhanced NLRP3 inflammasome activation and interleukin-1β (IL-1β) production after an ischemic stroke. A worsening of the neurological prognosis is brought on by cytotoxic swelling and edema production caused by impaired cell volume regulation. These conditions cause a marked increase in brain damage, inflammation, and cerebral edema (Tóth et al., 2022).

#### 3.1.6 Microglia/macrophages: innate immune response in STAT6-KO mice following ischemic stroke

Signal transduction and transcriptional activator proteins, known as STATs, constitute a unique family of proteins capable of binding to DNA. The functional state of macrophages and microglia is regulated by multiple members of the STAT family. In particular, STAT6 signaling causes macrophages to exhibit an anti-inflammatory phenotype, whereas STAT1 activation encourages inflammatory activity. Moreover, IL-4, a STAT6 activator, can direct macrophages and microglia toward a favorable phenotype, aiding in the recovery process following a brain ischemic stroke (Cai et al., 2019).

After an ischemic stroke, microglia and macrophages’ STAT6 is activated. This activation modifies the microglia and macrophage phenotype via the STAT6/Arg1 pathway, accelerating the resolution of inflammation, enhancing phagocytosis, and ultimately improving the prognosis for stroke patients. The absence of STAT6 leads to impaired phagocytosis, exacerbated inflammation, and increased ischemic brain damage (Cai et al., 2019).

#### 3.1.7 Microglia: innate immune response in microglial HDAC3 CKO mice following ischemic stroke

Recovery from a stroke and cerebral ischemia–reperfusion (I/R) injuries are both impacted by histone deacetylases (HDACs). After I/R, oxidative stress activates HDAC2, and blocking HDAC2 can improve cellular survival and neurogenesis after stroke. Ischemic preconditioning causes neurons’ HDAC3 to be degraded, which increases the neurons’ resistance to I/R. In neurons, inhibiting HDAC1 and HDAC6 can increase cellular viability while inhibiting HDAC4 and HDAC5 can decrease it (Liao et al., 2020).

After an ischemic stroke, HDAC3 regulates the nuclear localization and acetylation of microglial p65, which enhances the activation of the cGAS-STING pathway and transcriptionally promotes the production of cGAS. This process aids in the development of a pro-inflammatory milieu. The cGAS-STING pathway is less activated, inflammatory mediators are secreted less frequently, the inflammatory response is lessened, and the neurological and functional prognosis is improved in microglial HDAC3 CKO mice (Liao et al., 2020).

#### 3.1.8 Neutrophils: innate immune response in neutrophil α9^−/−^ mice following ischemic stroke

Integrin α9β1 upregulates neutrophil activation and works in concert with β2 integrins to stabilize neutrophil adhesion to activated endothelium. β1 is the sole partner for α9. Besides neutrophils, α9β1 is expressed in other cell types, including monocytes, smooth muscle cells, hepatocytes, endothelial cells, and epithelial cells (Patel et al., 2023).

The deficiency of neutrophil *α*9 limits the formation of ischemic thrombi associated with reduced fibrin deposition and platelet aggregation in the brain. When an ischemic stroke occurs, the lack of α9 in neutrophils limits the inflammatory response by lowering TNF-α, IL-1β, and IL-6 levels (Patel et al., 2023). Neutrophil α9^−/−^ mice after an ischemic stroke have a decreased thrombotic inflammatory response, which improves their neurological and functional results.

#### 3.1.9 Mononuclear phagocytes: innate immune response in bone marrow-specific pyruvate kinase M2 CKO mice following ischemic stroke

The glycolytic enzyme pyruvate kinase muscle 2 (PKM2) is an important regulator of aerobic glycolysis and also functions as an activator of transcription for pro-inflammatory cytokines such as interleukin-1 beta (IL-1β) and IL-6 (Dhanesha et al., 2022). Mammals have four different isoforms of pyruvate kinase (PK) (PKR, PKL, PKM1, PKM2), which are encoded by two separate genes, PKLR and PKM (Dhanesha et al., 2022).

PKM2 promotes STAT3 phosphorylation, which in turn controls peripheral neutrophil post-ischemic inflammation after an ischemic stroke. In bone marrow-specific PKM2^−/−^ mice, there is a reduction in fibrinogen, platelet deposition, neutrophil infiltration, and inflammatory cytokines in the brain, leading to improved neurological and functional outcomes (Dhanesha et al., 2022).

#### 3.1.10 Mononuclear phagocytes: innate immune response in myeloid cell autophagy-deficient (Atg5flox/flox LysMCre+) Atg5 KO mice following ischemic stroke

When it comes to ischemic brain injury, inflammatory cells have both beneficial and harmful functions. Myeloid cells’ autophagy, particularly that of macrophages, can reduce oxidative stress, stop the overproduction of pro-inflammatory cytokines, and stop the activation of inflammatory bodies (Kotoda et al., 2018).

Following ischemic stroke, autophagy can suppress the overall activity of inflammatory bodies by effectively removing endogenous inflammatory body activators, including reactive oxygen species and damaged mitochondrial DNA, thereby reducing secondary injury mediated by sustained and aberrant inflammation in the penumbra region (Patel et al., 2023). Mice with impaired myeloid cell autophagy (Atg5flox/flox LysMCre+) show increased levels of pro-inflammatory cytokines, which exacerbate ischemic brain damage by causing excessive inflammation in the subacute and chronic phases after ischemia brain injury (Kotoda et al., 2018; Wu et al., 2023).

#### 3.1.11 Mast cells: innate immune response in C-kit^−/−^ MC KO mice following ischemic stroke

Mast cells (MCs) are derived from hematopoiesis and are present in most tissues, with the lungs and intestines being the primary sites of their distribution. MCs serve as the initial line of defense against viruses and injuries, as well as environmental antigens and allergens. MCs release preformed and newly synthesized mediators, notably histamine (HA). HA is a neurotransmitter that promotes inflammation and is essential for the maturation of MC progenitor cells (Conesa et al., 2023).

Mast cells (MCs) react quickly to damage after an ischemic stroke and release histamine (HA). The main location of HA synthesis is the gastrointestinal system, and MCs are transported from the gut to the brain. Increased recruitment of MCs in the ischemic brain leads to neuroinflammation. In C-kit^−/−^ MC KO mice, the release of histamine can be prevented, which can alleviate neuroinflammation following ischemic stroke and improve neurological and functional outcomes (Conesa et al., 2023).

We examine how particular innate immune cells affect the response after an ischemic stroke in this section. Notably, animals with conditional deletion of the IRF5 and IRF4 genes in microglia following stroke showed clear behavioral changes. While mice with IRF4 CKO showed an increased M1-type pro-inflammatory response, mice with IRF5 CKO (conditional knockout) showed an improved M2-type anti-inflammatory response. These results imply that the inflammatory response and post-stroke recovery are strongly influenced by the activation state of microglia. Additionally, we observed that vitamin D signaling affects neuroinflammation and blood-brain barrier integrity in VDR CKO mice, and that TREM2-KO mice play a role in removing cellular debris and regulating anti-inflammatory responses. These findings highlight the possibility of treatment approaches that target particular innate immune cells, potentially enhancing stroke recovery.

### 3.2 Influencing the innate immune response following ischemic stroke through pattern recognition receptors

#### 3.2.1 TLR2/4: innate immune response in TLR2/4 mice following ischemic stroke

The family of pattern recognition receptors (PRRs) known as toll-like receptors (TLRs) is able to recognize pathogen-associated molecular patterns (PAMPs) from microbes or damage-associated molecular patterns (DAMPs) generated from injured tissues (Tajalli-Nezhad et al., 2019). These receptors, by sensing different stimuli, recruit specific adapter proteins, activate a series of signal transduction cascades, and trigger specific immune responses, serving as a bridge between innate immunity and adaptive immune responses.

When TLR2/4 binds to the endogenous ligand HMGB1, it triggers a sequence of signal transduction cascades that result in the production of inflammatory cytokines such TNF-*α*, IL-1β, and IL-6, which exacerbates cerebral ischemia–reperfusion injury. Compared to WT mice, TLR2/4-deficient mice exhibit lower levels of these inflammatory cytokines, smaller infarct volumes, milder neuroinflammation, and higher neuronal survival rates in the ischemic hemisphere after cerebral ischemia–reperfusion injury (Tajalli-Nezhad et al., 2019; Qiu et al., 2019).

We specifically looked at the activity of Toll-like receptors (TLRs) when investigating the role of pattern recognition receptors in the innate immune response after stroke. TLRs are essential for controlling the inflammatory response following ischemic stroke, as evidenced by the reduced levels of inflammatory cytokines and smaller infarct volumes seen in mice with genetic deletion of TLR2/4. By modifying TLR activity, these findings offer a scientific basis for the creation of innovative treatment approaches that target TLRs and may lessen brain damage after stroke.

### 3.3 Influencing the innate immune response following ischemic stroke through signaling pathways

#### 3.3.1 Signaling pathway intermediates (TRAF6): innate immune response in *β*-arrestin 2 KO (ARRB2^−/−^) mice following ischemic stroke

G protein-coupled receptors (GPCRs) are desensitized and internalized by adapter proteins called beta-arrestins, which stops G protein activation. They have been identified as scaffold proteins that regulate a wide range of biological processes by binding to different target molecules. The regulating function of beta-arrestin 2 (ARRB2) in inflammatory reactions is crucial (Chen et al., 2019).

Following cerebral ischemia–reperfusion (I/R) injury, ARRB2 negatively regulates the NF-κB inflammatory signaling triggered by NOD2 (nucleotide-binding oligomerization domain-containing protein 2) through its interaction with TRAF6 in microglia (Chen et al., 2019). When ischemia–reperfusion (I/R) injury occurs in *β*-arrestin 2 knockout (KO) mice, the lack of ARRB2 results in worsened outcomes of ischemia–reperfusion stroke generated by NOD2 stimulation.

#### 3.3.2 Signaling pathway intermediates (MAPK): innate immune response in Gpx1 KO mice following ischemic stroke

A family of selenoenzymes known as glutathione peroxidases (Gpx) catalyzes the reduction of certain biological peroxides at the expense of reduced glutathione. Gpx1 is the most prevalent isoform, and it has been linked to neurodegenerative conditions such traumatic brain injury, Lewy body dementia, and Parkinson’s disease (Chen et al., 2011).

In order to shield the brain from oxidative stress and inflammation brought on by ischemia–reperfusion injury, Gpx1 performs a crucial regulatory role. The activation of pro-apoptotic p53 and Fas ligand (CD95/Apo1)-mediated pathways, a decrease in the Nrf2 antioxidant cascade, malfunctioning of the ubiquitin-proteasome system (UPS), and inhibition of the mitogen-activated protein kinase (MAPK) signaling pathway are observed in Gpx1 knockout (KO) mice after ischemic stroke. These findings exacerbate ischemic brain injury (Chen et al., 2011).

#### 3.3.3 Signaling pathway intermediates (TAK1): innate immune response in USP10 KO mice following ischemic stroke

A member of the cysteine protease ubiquitin-specific protease family, ubiquitin-specific protease 10 (USP10) possesses the enzymatic ability to cleave ubiquitin from ubiquitin-conjugated protein substrates. Key signaling pathways, including those involving p53 and PTEN, that are involved in cell proliferation, apoptosis, inflammation, autophagy, and tumor metabolism are modulated by USP10 (Wang et al., 2019). The absence of USP10 leads to a significant increase in the activation of NF-κB (Wang et al., 2019).

By directly interacting with Transforming Growth Factor *β*-Activated Kinase 1 (TAK1), USP10 protects against cerebral I/R injury after ischemic stroke. USP10 KO mice show accelerated NFκB signaling pathway, increased inflammatory response in the ischemic cortex’s penumbra area, marked apoptotic induction, and worsened ischemia brain injury in comparison to WT mice (Wang et al., 2019).

#### 3.3.4 Signaling pathway intermediates (TAK1): innate immune response in TAK1 mKO mice following ischemic stroke

Transforming Growth Factor *β*-Activated Kinase 1 (TAK1), also known as MAP3K7 (Mitogen-Activated Protein Kinase Kinase Kinase 7), serves a dual role as a regulator of cell death inducers and a variety of inflammatory signaling molecules. It includes key regulatory functions for IKK (I-κB Kinase), MAP2Ks, MKK3/6, MKK4/7, and AMPK (AMP-Activated Protein Kinase) (Wang et al., 2020).

Following cerebral ischemia–reperfusion, TAK1 plays a crucial role in the pro-inflammatory responses of macrophages (MΦ) and microglia (Mi). Pro-inflammatory genes and other myeloid cell activity-related functions in macrophages and microglia are downregulated or repressed in TAK1 Mi/MΦ-specific knockout mice. The specific conditional deletion of TAK1 in adult Mi/MΦ alleviates the pro-inflammatory response after ischemic stroke and improves long-term neurological function (Wang et al., 2020).

We examine how signaling pathways affect the innate immune response after an ischemic stroke in this section. In particular, we found that β-arrestin 2 knockout (KO) mice have worse stroke outcomes following ischemia-reperfusion injury because NOD2 stimulation exacerbates activation of the NF-κB inflammatory signaling pathway. These results demonstrate the critical function of β-arrestin 2 in controlling inflammatory reactions and could offer hints for the creation of novel treatment approaches.

### 3.4 Influencing the innate immune response following ischemic stroke through innate immune molecules

#### 3.4.1 Cytokines: innate immune response in MCP-1^−/−^ mice following ischemic stroke

One of the most important chemokines produced by neurons, astrocytes, and endothelial cells in response to hypoxia is monocyte chemoattractant protein-1 (MCP-1), commonly referred to as CCL2. It acts as the main chemoattractant, luring neutrophils, macrophages, and monocytes toward the center of the infarct (Strecker et al., 2013).

Following ischemic stroke, MCP-1−/− mice exhibit reduced expression of IL-1β, IL-6, and G-CSF, which helps prevent further damage to the blood–brain barrier in mice. Reductions in the infiltration of neutrophils, macrophages, and monocytes into the ischemic brain tissue result in better neurological and functional outcomes as well as a decrease in local inflammatory responses (Strecker et al., 2013).

#### 3.4.2 Cytokines: innate immune response in IL-1R1 ^−/−^ mice following ischemic stroke

A pro-inflammatory cytokine called interleukin-1 (IL-1) has been linked to a number of neurological illnesses, including ischemic stroke. The IL-1 type I receptor (IL-1R1), which is known to transduce intracellular signals, and the IL-1 type II receptor, which does not directly signal, are the two types of IL-1 receptors that have been found to date (Lazovic et al., 2005).

Due to the elimination of IL-1R1 signaling, IL-1R1 ^−/−^ mice can preserve energy status, interfere directly with the cytokine activation of immune cells, limit the damage caused by free radicals mediated by inducible nitric oxide synthase (iNOS), and disrupt the production of chemokines after an ischemic stroke. This results in better neurological and functional outcomes and less brain damage after an ischemic stroke (Lazovic et al., 2005).

#### 3.4.3 Complement: innate immune response in MBL^−/−^ mice following ischemic stroke

The pathogenesis of stroke involves the complement system, which is a powerful inducer of inflammatory reactions. Brain damage progression is largely influenced by the lectin pathway (LP), one of the complement system’s activation routes. Mannose-binding lectin (MBL), one of the LP’s starting components, can be targeted in mice to reduce damage through pharmacological suppression or genetic deficit (Orsini et al., 2018).

Following ischemic stroke, circulating mannose-binding lectin MBL drives platelet activation. Activated platelets release interleukin-1 alpha (IL-1α), which upon binding to its receptor (IL-1R1) induces vascular inflammation and promotes vascular injury (Orsini et al., 2018). Endothelial injury results from the deposition of mannose-binding lectin (MBL) by damaged endothelial cells. This leads to complement activation and pro-inflammatory activation of the endothelium, including the upregulation of intercellular adhesion molecule-1 (ICAM-1) (Orsini et al., 2018). Following ischemic stroke, MBL^−/−^ mice exhibit reduced levels of IL-1α, an attenuation of the inflammatory phenotype, and improved neurological and functional outcomes.

#### 3.4.4 Complement: innate immune response in C3 ^−/−^ mice following ischemic stroke

Neuronal damage results from inflammation after an experimental stroke. Through influencing the upregulation of adhesion molecules, neutrophil chemotaxis, platelet activation, and the generation of reactive oxygen species, the complement cascade plays a crucial role in both starting and controlling the inflammatory process (Mocco et al., 2006). After cerebral ischemia–reperfusion injury in adult mice, the complement system, which is mostly dependent on C3, mediates inflammatory neuronal damage (Mocco et al., 2006).

C3-deficient (C3 ^−/−^) mice show a substantial drop in infiltrative neutrophils and oxidative stress levels after an ischemic stroke, which diminishes brain damage and improves neurological and functional results (Mocco et al., 2006) (Table 1).

The function of innate immunity molecules in the innate immune response after stroke is examined in this section, with special focus on the outcomes of MCP-1 and IL-1R1 gene knockout mice. Following a stroke, MCP-1−/−mice show enhanced blood-brain barrier integrity and decreased production of inflammatory cytokines, whereas IL-1R1−/−mice show improved functional recovery and lower neuroinflammation. These findings raise the possibility of developing novel therapeutic approaches by indicating that modifying particular innate immune molecules can successfully control the inflammatory response after stroke. An alternate structure and a synopsis of the key points are shown in the following table.

**Table 1:** Gene table associated with innate immune response after ischemic stroke.

Summarize							
References	Target of genetic modification	Gene	KO/overexpersion/KI	Mouse models	Biomarkers	Mechanism	Outcome
	3.1 Modulating the Innate Immune Response Following Ischemic Stroke by Targeting Microglia, Neutrophils, Mononuclear Macrophages, and Mast Cells						
Al Mamun et al. (2020)	3.1.1 Microglia	IRF5, IRF4	CKO (Microglia)	tMCAO	IL-4, IL-10, IL-1β, TNFα, Pro-inflammatory mRNA levels (iNOS, TNFα, CD68, MHCII)	The absence of IRF5 signaling leads to an increase in IRF4 expression, enhanced activation of the M2 phenotype, and a reduction in pro-inflammatory responses. Conversely, the deficiency in IRF4 signaling results in increased IRF5 expression, heightened M1 activation, and intensified pro-inflammatory reactions.	Improvement in ischemic stroke/Deterioration in functional recovery
Sieber et al. (2013)	3.1.2 Microglia	TREM2	KO	tMCAO	Activation of microglia, TNFα, IL-1α, IL-1β, Chemotactic factor (CCL2, CCL3), Transcription of CX3CR1, Infiltration of CD3 positive T cells	Microglial activity is reduced, transcription of pro-inflammatory cytokines TNFα, IL-1α, and IL-1β is decreased, transcription of chemokines CCL2 (MCP1), CCL3 (MIP1α), and chemokine receptor CX3CR1 is reduced, followed by a decrease in the invasion of CD3-positive T cells.	Subacute inflammatory responses are attenuated without affecting the size of the ischemic stroke lesion
Cui et al. (2023)	3.1.3 Microglia/Macrophages	VDR	CKO (Microglia/Macrophages)	tMCAO	TNF-α, IFN-γ, CXCL10, T cells	Microglia/macrophages exhibit a more pronounced pro-inflammatory phenotype, secreting large amounts of TNF-α and IFN-γ, promoting the release of CXCL10 from endothelial cells, disrupting the blood-brain barrier, and enhancing the infiltration of peripheral T lymphocytes.	Exacerbating ischemic brain injury
Shi et al. (2011)	3.1.4 Microglia	NHE-1	KO	tMCAO	Activation of microglia, formation of astrocytes, TNF-α, IL-1β, IL-6	Microglial activation is reduced, activation of NADPH oxidase is diminished, and pro-inflammatory responses are decreased	Mitigates brain injury after ischemic stroke and improves neurological and functional prognosis
Tóth et al. (2022)	3.1.5 Microglia	NKCC1	CKO (Microglia)	tMCAO	Expression of inflammatory cytokines (G-CSF, IL-1α, IL-1β, NLRP3, IL-6)	Activation of the NLRP3 inflammasome and increased production of interleukin-1 beta (IL-1β), impaired cell volume regulation, and the formation of cytotoxic cellular swelling and edema	Exacerbates brain injury, inflammation, cerebral edema, and poor neurological prognosis
Cai et al. (2019)	3.1.6 Microglia	STAT6	KO	tMCAO	Microglia, macrophages, astrocytes, oligodendrocytes, cerebral blood flow, IL-6, TNF-α, IL-10	Impaired phagocytosis, exacerbated inflammation	Exacerbates ischemic brain injury
Liao et al. (2020)	3.1.7 Microglia	HDAC3	CKO (Microglia)	tMCAO	Expression of (IFN-β, IL-6) and protein levels of (pIRF3, cGAS, GAPDH).	The activation of the cGAS-STING pathway is reduced, the secretion of inflammatory mediators is decreased, and the inflammatory response is alleviated	Improves neurological and functional outcomes
Patel et al. (2023)	3.1.8 Neutrophils	Neutrophils α9	KO	tMCAO	Fibrin, neutrophils, p-NFκB, TNFα and IL-1β, markers of neutrophil extracellular traps (NETs)	Restricted the inflammatory response following the onset of ischemic stroke (reducing TNF-α, IL-1β, and IL-6)	Thrombotic inflammatory responses are alleviated, leading to improved neurological and functional outcomes
Dhanesha et al. (2022)	3.1.9 Monocytes/Macrophages	PKM2	CKO(Marrow cells)	tMCAO	TNF-α, IL-1β, IL-6, neutrophils, fibrinogen, platelets.	Fibrinogen, platelet deposition, neutrophil infiltration, and a reduction in inflammatory cytokines	Improving neurological and functional outcomes
Kotoda et al. (2018) and Wu et al. (2023)	3.1.10 Monocytes/Macrophages	Atg5, floxed	KO	tMCAO	Expression levels of TNF-a, IL-1 β, IL-6, MM9, MCP-1, infiltration of macrophages, granulocytes, and microglia	Elevated levels of pro-inflammatory cytokines lead to excessive inflammation during the subacute and chronic phases following ischemic brain injury	Exacerbating ischemic brain injury
Conesa et al. (2023)	3.1.11 Mast cells	C-kit	CKO (Mast cells)	tMCAO	Mast cells, histamine, IL-6	Preventing the release of histamine from mast cells	Alleviate neuroinflammation after ischemic stroke, improving neurological and functional outcomes
	3.2 Influencing the Innate Immune Response Following Ischemic Stroke through Pattern Recognition Receptors (PRRs)						
Tajalli-Nezhad et al. (2019) and Qiu et al. (2019)	3.2.1 TLR2/4 signaling pathway	TLR2/4	KO	tMCAO	TNF-α, IL-1β, IL-6, iNOS, Expression of brain pathways (p-JNK, IkB-a, NF-kB)	The levels of inflammatory factors are reduced	Mild neuroinflammation and higher neuronal survival rates in the ischemic hemisphere
	3.3 Influencing the Innate Immune Response Following Ischemic Stroke through Signaling Pathways						
Chen et al. (2019)	3.3.1 Intermediate link in the signaling pathway (TRAF6)	ARRB2	KO	tMCAO	NF‐κB, IκBα, COX‐2, MMP‐2, MMP‐9	Aggravating the outcomes of ischemic stroke induced by NOD2 stimulation after cerebral ischemia/reperfusion (I/R) injury	Exacerbating ischemic brain injury
Chen et al. (2011)	3.3.2 Intermediate link in the signaling pathway (MAPK)	GPx1	Overexpersion	tMCAO	Gene expression of tumor necrosis factor, chemokine interleukin receptor, neutrophil cytoplasmic factor, toll like receptor	Reducing the activity of cells that produce inflammatory mediators and decreasing the number of injured brain cells significantly alleviates the inflammatory response following ischemia	Reduce brain damage after ischemic stroke and improve neurological and functional outcomes
Wang et al. (2019)	3.3.3 Intermediate link in the signaling pathway (TAK1)	USP10	KO	tMCAO	TNF-a, IL-1β, MCP1, CXCL1, Protein expression level (p-IKKb, p-IkBa, p-NF-kB, p-Mek1/2, p-ERK1/2, p-p38, p-JNK, p-c-JunS63, p-c-JunS73)	Accelerating the NF-κB signaling pathway, promoting inflammatory responses in the penumbra of cortical ischemia, and significantly inducing apoptosis	Exacerbating ischemic brain injury
Wang et al. (2020)	3.3.4 Intermediate link in the signaling pathway (TAK1)	TAK1	CKO (Microglia, Macrophages)	tMCAO	The quantity of macrophages, T cells, neutrophils, and microglia, the expression of pro-inflammatory genes	The pro-inflammatory genes and various functions related to the activity of myeloid cells are downregulated or suppressed in microglia and macrophages	Reduce the pro-inflammatory response after ischemic stroke and improve long-term neurological function
	3.4. Influencing the Innate Immune Response Following Ischemic Stroke through Innate Immune Molecules						
Strecker et al. (2013)	3.4.1 Cytokine	MCP-1	KO	tMCAO	Blood-brain barrier-related genes (occludin, occluden -1, occluden -2), IL-1β, IL-6, G-CSF	The expression of IL-1β, IL-6, and G-CSF is reduced, preventing further damage to the blood-brain barrier in mice, with decreased infiltration of monocytes, macrophages, and neutrophils in the ischemic brain tissue	Localized inflammatory responses are alleviated, improving neurological and functional outcomes
Lazovic et al. (2005)	3.4.2 Cytokine	IL-1R1	KO	tMCAO	The mRNA levels of inducible nitric oxide synthase (iNOS) and endothelial nitric oxide synthase (eNOS), leukocyte infiltration	Maintain energy status, disrupt the production of chemokines, directly interfere with the cytokine activation of immune cells, and limit the free radical damage mediated by inducible nitric oxide synthase (iNOS)	Mitigate brain damage following ischemic stroke and improve neurological and functional prognosis
Orsini et al. (2018)	3.4.3 Complement	MBL	KO	tMCAO	IL-1α, CXCL1, ICAM-1	Platelet activation is reduced, and the content of IL-1α is decreased	The inflammatory phenotype is mitigated, improving neurological and functional outcomes
Mocco et al. (2006)	3.4.3 Complement	C3	KO	tMCAO	Malondialdehyde, infiltrating granulocytes	Infiltrating neutrophils are significantly reduced, and levels of oxidative stress are decreased	Reduce brain injury after ischemic stroke and improve neurological and functional prognosis

## 4 Discussion

We have reviewed the literature from four perspectives in this review, which covers the innate immune response after ischemic stroke in transgenic mice: innate immune cells, receptors, signaling pathways, and innate immune chemicals.

We found that conditionally knocking out genes in microglia or macrophages of mice can intuitively alter their pro-inflammatory/anti-inflammatory phenotype, affect the expression of their associated inflammatory factors, and further influence the prognosis of stroke (Shi et al., 2011; Tóth et al., 2022; Cai et al., 2019; Liao et al., 2020; Patel et al., 2023; Dhanesha et al., 2022; Kotoda et al., 2018; Wu et al., 2023; Conesa et al., 2023; Tajalli-Nezhad et al., 2019; Qiu et al., 2019). For example: Microglial IRF5 CKO mice and microglial IRF4 CKO mice directly alter the activation phenotype of microglia, leading to corresponding changes in inflammatory responses, which in turn affect the prognosis of stroke (Al Mamun et al., 2020). For instance: The autophagy failure in myeloid cells, particularly macrophages, in mice with myeloid cell autophagy insufficiency, is unable to efficiently eliminate endogenous inflammatory body activators. This aggravates ischemic stroke by causing an overabundance of pro-inflammatory cytokines to be released, oxidative stress, and the activation of inflammatory bodies (Kotoda et al., 2018). According to the text, local inflammation following stroke is also directly impacted by the deletion of genes linked to innate immune components in mice. For example: MCP-1^−/−^ mice can affect the release of IL-1α, reducing inflammatory responses. However, their limitations are also quite evident, as they can only affect some inflammatory mediators and are not as comprehensive as selectively knockout mice (Strecker et al., 2013). According to the text, the expression of inflammatory factors following a stroke in mice is more indirectly affected by the knockout of genes linked to innate immune receptors and signaling pathways. These mechanisms are complex and can be challenging to control, and the effects are much smaller than in mice with conditional knockouts. For example, in the classic TLR2/4 mice, after ischemic stroke, TLR2/4 binds to the endogenous ligand HMGB1, activating a series of signal transduction cascades, which then produce pro-inflammatory cytokines affecting the prognosis of ischemic stroke (Tajalli-Nezhad et al., 2019; Qiu et al., 2019). The signaling pathways are intricate and complex, and targeting a specific pathway has a very limited impact on the organism.

Current research still has its limitations. Future research on genetically modified mice should be more committed to using conditional knockout mice. In particular, genetically modified mice targeting microglia and mononuclear/macrophage cells will yield greater benefits. Following cerebral ischemic stroke, microglia and mononuclear/macrophage cells play a crucial role in the innate immune response (Xiong et al., 2016; Li et al., 2021; Lee et al., 2004; Colton, 2009; Price et al., 2004; Garcia-Bonilla et al., 2014; Gidday et al., 2005; Gelderblom et al., 2009; Jin et al., 2009), and perhaps this presents a new avenue for therapeutic intervention. Altering their pro-inflammatory phenotype has consistently been a focal point of current research. The genetically modified mice studies summarized in this article, especially focusing on genetically modified mice targeting microglia and mononuclear/macrophage cells in ischemic stroke, are conducive to exploring and developing drugs related to the mechanisms for future clinical treatment.

The main areas of current study lacking are a thorough understanding of the dynamic changes that occur in innate immunity cells after a stroke and how to precisely manipulate these cells’ activation states in order to facilitate brain healing. The following directions should be the main areas of future research:

1. Precise Cell Marking and Tracking Technologies: Creating novel genetic tagging instruments that provide accurate monitoring and control of macrophage and microglia post-stroke.
2. Single-Cell Analysis Technologies: Revealing cellular heterogeneity across various brain regions and time points using technologies like single-cell RNA sequencing, which serves as a foundation for precision medicine.
3. Clinical Translational Research: Investigating how immune modulation pathways found in animal models may be applied to human stroke, including the planning and execution of therapeutic trials.
4. Interdisciplinary Collaboration: Close cooperation between immunologists, doctors, neuroscientists, and bioinformaticians will help to gain a multifaceted understanding of the immunological mechanisms underlying stroke.

In addition, our review offers a revised viewpoint based on current knowledge, highlighting in particular the crucial function that signaling pathways and pattern recognition receptors play in controlling the innate immune response. We think that by focusing on these chemicals, novel treatment approaches to lessen inflammatory reactions and brain damage after stroke may be developed.

Varied transgenic mice models have varied benefits and drawbacks when it comes to studying immunological responses. As an illustration:

1. While models of microglial IRF5 and IRF4 CKO mice are particularly helpful in examining the transition between microglial activation states, they might not accurately replicate the intricacy of a real stroke.
2. While TREM2-KO mouse models are excellent for examining the role of cellular debris clearance and anti-inflammatory responses, they may not fully account for the contributions of other types of immune cells.
3. Models of myeloid cell autophagy-deficient mice demonstrate the significance of autophagy in controlling inflammatory responses; nevertheless, due to the intricacy of the autophagy mechanism, research results may differ.
4. Researchers should take into account the specific objectives of the study as well as the models’ specialized applicability while choosing models. For example, global knockout models might be required to examine the systemic impact of inflammatory cytokines, but microglia-specific knockout mice might be more appropriate to study the involvement of microglia after stroke.

The literature does contain some inconsistent findings, which could be the consequence of variations in the experimental setup, different mice strains, or genetic alterations. For instance, the research of immunological responses may be impacted by the varying susceptibilities to stroke of different mouse strains employed in different laboratories. Additionally, distinct patterns of brain injury and ensuing immune responses can result from the stroke model preparation technique (e.g., photothrombosis or the filament method). As a result, when evaluating these distinctions and planning research, we must use caution.

We think that a better knowledge of the function of innate immunity after stroke will contribute to the creation of more potent therapeutic approaches.
